# A study of virulence and antimicrobial resistance pattern in diarrhoeagenic *Escherichia coli* isolated from diarrhoeal stool specimens from children and adults in a tertiary hospital, Puducherry, India

**DOI:** 10.1186/s41043-018-0147-z

**Published:** 2018-07-13

**Authors:** Mailan Natarajan, Deepika Kumar, Jharna Mandal, Niranjan Biswal, Selvaraj Stephen

**Affiliations:** 1 Department of Microbiology, Jawaharlal Institute of Postgraduate Medical Education and Research (JIPMER), Puducherry, 605006 India; 2 Department of Paediatrics, Jawaharlal Institute of Postgraduate Medical Education and Research (JIPMER), Puducherry, 605006 India; 3 Department of Microbiology, Mahatma Gandhi Medical College and Research Institute (MGMC & RI), Puducherry, 607 402 India

**Keywords:** Diarrhoeagenic *Escherichia coli* (DEC), Diarrhea, Children, Adults, Antimicrobial resistance (AMR) genes

## Abstract

**Background:**

Emergence of atypical enteropathogenic *Escherichia coli* (EPEC) and hybrid *E. coli* (harboring genes of more than one DEC pathotypes) strains have complicated the issue of growing antibiotic resistance in diarrhoeagenic *Escherichia coli* (DEC). This ongoing evolution occurs in nature predominantly via horizontal gene transfers involving the mobile genetic elements like integrons notably class 1 integron. This study was undertaken to determine the virulence pattern and antibiotic resistance among the circulating DEC strains in a tertiary care center in south of India.

**Methods:**

Diarrhoeal stool specimens were obtained from 120 children (< 5 years) and 100 adults (> 18 years), subjected to culture and isolation of diarrhoeal pathogens. Conventional PCR was performed to detect 10 virulence and 27 antimicrobial resistance (AMR) genes among the *E. coli* isolated.

**Results:**

DEC infection was observed in 45 (37.5%) children and 18 (18%) adults, among which [18 (40%), 10 (10%)] atypical EPEC was most commonly detected followed by [6 (13.3%), 4 (4%)] ETEC, [5 (11.1%) 2 (2%)] EAEC, [(3 (6.6%), 0 (0%)] EIEC, [3 (6.6%), 0 (0%] typical EPEC, and [4 (8.8%), 1 (1%)] STEC, and no NTEC and CDEC was detected. DEC co-infection in 3 (6.6%) children, and 1(1%) adult and sole hybrid DEC infection in 3 (6.6%) children was detected. The distribution of sulphonamide resistance genes (*sulI*, *sulII*, and *sulIII* were 83.3 and 21%, 60.41 and 42.1%, and 12.5 and 26.3%, respectively) and class 1 integron (*int1*) genes (41.6 and 26.31%) was higher in DEC strains isolated from children and adults, respectively. Other AMR genes detected were *qnrS*, *qnrB*, *aac(6’)Ib-cr*, *dhfr1*, *aadB*, *aac(3)-IV*, *tetA*, *tetB*, *tetD*, *catI*, *blaCTX*, *blaSHV*, and *blaTEM.* None harbored *qnrA*, *qnrC*, *qepA*, *tetE*, *tetC*, *tetY*, *ermA*, *mcr1*, *int2*, and *int3* genes.

**Conclusions:**

Atypical EPEC was a primary etiological agent of diarrhea in children and adults among the DEC pathotypes. Detection of high numbers of AMR genes and class 1 integron genes indicate the importance of mobile genetic elements in spreading of multidrug resistance genes among these strains.

**Electronic supplementary material:**

The online version of this article (10.1186/s41043-018-0147-z) contains supplementary material, which is available to authorized users.

## Background

Diarrhoeal disease is a major cause of morbidity in all age groups, and it is the second most important cause of mortality in children less than 5 years of age. Though being treatable, burden accounts for 1700 million cases of diarrhea and 5,000,000 deaths among children annually [1]. According to recent data, 10–13 children under 5 years of age die every 1 h due to diarrhea in India [2]. A high incidence of diarrhoeal diseases has also been documented in adults [3]. One of the most common bacterial agents of infective diarrhea is *Escherichia coli*. Few studies conducted in India have documented a high prevalence of diarrhoeagenic *Escherichia coli* (DEC) in children and adults [4]. Most strains of *E. coli* are intestinal commensal flora of human, birds, and animals. While few strains can cause a variety of intestinal diseases like food poisoning, abdominal cramping, pain or tenderness, nausea, vomiting, and diarrhea both in human and animals, the severity of infection in the host ranges from mild to life-threatening.

Based on the virulence factors, pathogenicity and clinical manifestation *E. coli* strains are categorized into five pathotypes, namely enterotoxigenic *E. coli* (ETEC), enteropathogenic *E. coli* (EPEC), Shiga toxin-producing *E. coli* (STEC), Enteroinvasive *E. coli* (EIEC), and Enteroaggregative *E. coli* (EAEC), which are collectively termed as DEC and have been associated with worldwide outbreaks since 1952 in children, adolescents, and adults [5, 6]. Recent studies from Africa, Spain, India, China, Mexico, and Japan have documented evolving variants like atypical and hybrid strains of DEC where they also noticed an increase in the severity of infection when compared to infection caused by typical DEC strains [4].

Apart from these DEC pathotypes, few extraintestinal *E. coli* were also responsible for intestinal infection in humans and animals like cell-detaching *E. coli* (CDEC) and necrotoxic *E. coli* (NTEC) which are known to produce cytotoxic necrotizing factors (*CFN 1*&*2*). Small outbreaks have been reported by these pathotypes in a few places [7, 8].

In view of increased consumption of prophylactic antibiotic agents by international travelers, selective pressure by these allows the resistant organism to survive and propagation of antimicrobial resistance (AMR) genes among these strains which is primarily due to horizontal gene transfer (HGT) [9, 10]. Worldwide studies have documented the high prevalence and varying pattern of AMR in DEC [11–16]. On the other hand, there is inadequate information concerning AMR pattern and the molecular resistance mechanisms in DEC from our region. Hence, this study was undertaken to look for DEC and its variants as well as to study the AMR mechanisms operating in them.

## Methods

### Study design and site

This cross-sectional study was carried out from July 2015 to June 2016 on diarrhoeal stool specimens from children (less than 5 years of age) and adults (more than 18 years of age) at JIPMER (a tertiary care public hospital cum research institute) situated in Pondicherry, India.

### Specimens and bacterial isolates

A total of 220 consecutive diarrhoeal stool specimens were collected from 120 children and 100 adults. The samples were inoculated on to MacConkey agar (MAC) from which three to five colonies were picked and inoculated into 2 ml Luria-Bertani (LB) broth; the isolates were stored in LB with 50% of glycerol and stored in − 80 °C till further use. DNA was extracted from the *E. coli* isolates using QIAamp DNA Mini Kit (Qiagen, Hilden, Germany). Stool specimens was subjected to culture for the detection of other bacterial agents causing diarrhea using standard protocol on to MAC, xylose lysine deoxycholate (XLD) agar, and thiosulfate-citrate-bile salt-sucrose TCBS agar for the detection of *Shigella* spp., *Aeromonas* spp*.*, *Salmonella* spp., and *Vibrio* spp. Subculture was done post enrichment in selenite F broth and alkaline peptone water onto MAC, XLD, and TCBS, respectively [17].

### Detection of virulence genes and AMR genes in DEC

Conventional PCR assay was used to detect 7 DEC pathogroup using 10 specific virulence genes as mentioned in Additional file 1: Tables S1 and S2. AMR genes (plasmid-borne) belonging to nine antimicrobial families and integron genes belonging to three classes of integrons were studied for all the confirmed DEC as mentioned in Additional file 1: Tables S1 and S2. The details of targeted genes, primer sequence, amplicon base pair size, reaction volume, and thermocycling conditions used as mentioned in Additional file 1: Tables S1 and S2. PCR was carried out in thermal cycler Eppendorf Mastercycler Nexus (Eppendorf, Hamburg, Germany).

### Gel electrophoresis

Gel electrophoresis was performed using 1.5% agarose (Sigma-Aldrich, USA) gel stained with ethidium bromide; 100 bp DNA Ladder (Genei Laboratories Pvt., Ltd., India) was used to measure the size of the amplicons (base pairs). Separated PCR products were visualized by Gel Doc XR System, Bio-Rad, Hercules, California, USA.

### Antimicrobial susceptibility testing

DEC isolates were subjected to antimicrobial susceptibility testing. The disk diffusion test was performed according to Clinical and Laboratory Standards Institute guidelines for amikacin (30 μg/disk), gentamicin (10 μg/disk), ciprofloxacin (5 μg/disk), levofloxacin (5 μg/disk), tetracycline (30 μg/disk), chloramphenicol (30 μg/disk), co-trimoxazole (1.25/23.75 μg/disk), cefoperazone-sulbactam (75/30 μg/disk), ceftazidime (30 μg/disk), and ceftriaxone (30 μg/disk); all these disks were procured from Bio-Rad, USA. ATCC 25922 (*E. coli*) was used as a quality control for antimicrobial susceptibility testing [18].

### Statistical analysis

All categorical variables were expressed as percentages (%). Chi-square test was used to find the association between categorical variables. Fisher’s exact test was used wherever appropriate. Likelihood of finding AMR genes in DEC pathotypes harboring was calculated using odds ratio (OR) and 95% confidence intervals (CIs), a *p* value of < 0.05 was considered statistically significant. Statistical analysis was performed using Epidata Analysis V2.2.3.187 and OpenEpi Version 3.01.

### Scientific and ethical assertion

This study was approved by the JIPMER Scientific Advisory Committee (project no. JSAC 20/5/2015) and JIPMER Institute Ethics Committee for Human studies (project no. JIP/IEC/2015/15/743).

### Gene sequencing

Sequencing was performed at BioServe Biotechnologies Pvt., Ltd. (Hyderabad, India). Aligned sequence (both forward and reverse) was searched in NCBI-BLAST (megablast) for the similarity of significant matches in the database. All the high similar nucleotide sequences were submitted to GenBank (NCBI).

## Results

### Prevalence of DEC pathogroups and other enteric bacterial pathogens in children and adults

Totally, 220 diarrhoeal stool specimens from children (*n* = 120) and adults (*n* = 100) were recruited in the study. Of the 120 children, 37.5% (*n* = 45, *p*-value = 0.01) and among the adults (*n* = 100), 18% (*n* = 18, *p* value = 0.01) presented with diarrhea were found to be positive for one or more pathotype of DEC (Table 1). DEC co-infection was observed in 6.6% (*n* = 3) of 45 DEC-infected children. While in adults 1% (*n* = 1) co-infection was observed with a hybrid DEC strain containing genes of both EPEC (*eaeA* and *bfpA*) and ETEC (*lt*) along with atypical EPEC (only *bfpA* gene was present). Apart from single and co-infection, 6.6% (*n* = 3) children had hybrid DEC infection. In adults, no EIEC could detect. No CDEC and NTEC (*CFN1* and *CFN2*) were detected in children and adults.

**Table 1:** Age groups and gender wise distribution of DEC pathotypes in children and adults

DEC infection types	DEC pathotypes	Genes	DEC infection in children (*n* = 45)							DEC infection in adults (*n* = 18)					
			Gender		Age in months (median* (IQR) − 12 (5.25–24))					Gender		Age in years (mean [± SD] − 44.74 [±15.24])			
			Male	Female	0–12	13–24	25–36	37–48	49–60	Male	Female	18–24	25–36	37–48	49–85
Single DEC infection	Typical EPEC	*eaeA* and *bfpA*	2	1	2	1	0	0	0	0	0	0	0	0	0
	Atypical EPEC	*eaeA*	3	2	2	2	1	0	0	1	3	0	1	1	2
		*bfpA*	5	8	7	6	0	0	0	5	1	0	1	3	2
	ETEC	*lt&st*	0	0	0	0	0	0	0	3	0	0	1	1	1
		*lt*	2	4	3	3	0	0	0	0	0	0	0	0	0
		*st*	0	0	0	0	0	0	0	0	1	0	0	0	1
	EAEC	*aggR*	3	2	2	3	0	0	0	1	1	0	2	0	0
	EIEC	*ial*	0	3	1	2	0	0	0	0	0	0	0	0	0
	STEC	*stx1*	4	0	2	2	0	0	0	1	0	1	0	0	0
DEC Co-infection	Atypical EPEC + Atypical EPEC	*eaeA* + *bfpA*	1	0	0	1	0	0	0	0	0	0	0	0	0
	EAEC + EIEC	*aggR* + *ial*	1	0	0	0	0	0	1	0	0	0	0	0	0
	Atypical EPEC + STEC	*bfpA* + *stx1*	1	0	0	0	0	0	1	0	0	0	0	0	0
	EPEC/ETEC (Hybrid DEC Strain) + atypical EPEC	*eaeA*/*bfpA/lt* + *bfpA*	0	0	0	0	0	0	0	1	0	0	0	0	1
Hybrid DEC infection	EAEC/EPEC	*aggR/bfpA*	2	0	2	0	0	0	0	0	0	0	0	0	0
	EAEC/EIEC	*stx1*/*ial*	1	0	0	0	0	1	0	0	0	0	0	0	0
Total			25	20	21	20	1	1	2	12	6	1	5	5	7
			(*n* = 45; *p* value ≤ 0.01)							(*n* = 18; *p* value ≤ 0.01)					

*DEC* diarrhoeagenic *E. coli*, *EPEC* enteropathogenic *E. coli*, *ETEC* enterotoxigenic *E. coli*, *EAEC* enteroaggregative *E. coli*, *STEC* Shiga toxin-producing *E. coli*, *EIEC* enteroinvasive *E. coli*, *NTEC* necrotoxigenic *E. coli*, *CDEC* cell-detaching *E. coli*, *IQR* interquartile range, *SD* stranded deviation

*Age expressed in median (non-normally distribution)

Other enteric bacterial pathogens detected in children and adults were *Shigella* spp. [*n* = 7 (5.8%) and *n* = 4 (4%)], *Aeromonas spp.* [*n* = 1 (0.83%) and *n* = 1 (1%)], and *Salmonella* spp. [*n* = 2 (2%) detected only in adults].

### Distribution of DEC according to age and gender (Table 1)

Of the 120 children, 63% (*n* = 76) were males and 37% (*n* = 44) were females, and among the 100 adults, 57% (*n* = 57) were males and 43% (*n* = 43) were females (Table 1).

Out of 120 children, 51.6% were less than 12 months of age (Additional file 2: Table S3). While in adults, 44% were between 49 and 85 years (Additional file 2: Table S4). The proportion of DEC infections among these groups was more in children than in adults. EPEC was detected more in children less than 12 months of age followed by ETEC. The likelihood of finding EPEC (*n* = 21) was 5.1 times (OR 5.1; 95% CI 1.478–23.72, *p* ≤ 0.05) more than that of ETEC (*n* = 6). In adults, the likelihood of finding atypical EPEC (*n* = 10) was 2.7 times (OR 2.7; 95% CI 0.8389–10.28, *p* ≥ 0.05) more than that of ETEC (*n* = 4). However, the likelihood of finding atypical EPEC (*n* = 10) was 5.4 times (OR 5.4; 95% CI 1.276–37.12, *p* ≤ 0.01) more than that the odds of finding EAEC (*n* = 2).

### Detection of AMR genes in DEC strains isolated from children and adults

All DEC strains isolated from children harbored one or more AMR gene. None of the isolates harbored *qnrA*, *qnrC*, *qepA*, *tetE*, *tetC*, *tetY*, *ermA*, and *mcr1* genes. Among the integron genes, the *int1* gene was found in 41.66% (*n* = 20) of the strains, while *int2* and *int3* were not detected in any of these strains (Tables 2 and 3).

**Table 2:** AMR and integron genes in DEC strains isolated from children and adults

AMR genes	AMR and integron genes in DEC strains with isolated from children											AMR and integron genes in DEC strains with isolated from adults						
	DEC single and co-infection							Hybrid DEC infection			Total	DEC single and co-infection					Hybrid infection	Total
	Typical EPEC	Atypical EPEC		ETEC	EIEC	EAEC	EHEC	EAEC + atypical EPEC		EHEC + EIEC		Atypical EPEC		ETEC	EAEC	EHEC	EPEC/ETEC (Hybrid)	
	*eaeA&bfpA*	*eaeA*	*bfpA*	*lt*	*ial*	*aggR*	*stx1*	*aggR/bfpA*	*aggR/bfpA*	*ial/stx1*		*eaeA*	*bfpA*	*eaeA/bfpA/lt + bfpA*	*aggR*	*stx1*	*eaeA/bfpA/lt*	
*qnrA*	0	0	0	0	0	0	0	0	0	0	0	0	0	0	0	0	0	0
*qnrB*	0	2	6	3	1	4	0	0	1	1	18	1	1	1	0	0	0	3
*qnrS*	2	3	6	3	2	3	1	0	1	0	21	0	1	2	0	0	0	3
*qnrC*	0	0	0	0	0	0	0	0	0	0	0	0	0	0	0	0	0	0
qeqA	0	0	0	0	0	0	0	0	0	0	0	0	0	0	0	0	0	0
*aac(6′)-Ib*	0	4	9	2	2	2	1	1	1	1	23	0	1	1	2	0	0	4
*sul1*	3	5	14	5	3	5	4	0	1	0	40	0	2	0	2	0	0	4
*sulII*	2	4	9	3	2	4	3	1	0	1	29	1	3	1	2	1	0	8
*sulIII*	0	1	3	1	0	0	0	0	0	1	6	1	1	1	1	0	0	4
*dhfr1*	3	1	6	3	2	4	2	1	1	1	24	0	1	0	2	0	0	3
*aac3*	0	2	2	2	1	1	0	0	0	0	8	0	0	0	0	0	0	0
*aadB*	0	0	1	1	0	1	1	0	0	0	4	0	0	1	0	0	0	1
*tetA*	1	1	4	0	1	0	0	1	1	1	10	0	2	2	0	0	0	4
*tetD*	0	1	1	0	2	1	1	0	0	0	6	0	0	0	0	0	0	0
*tetB*	1	2	4	2	0	1	1	0	1	0	12	1	1	0	1	0	0	3
*tetE*	0	0	0	0	0	0	0	0	0	0	0	0	0	0	0	0	0	0
*tetY*	0	0	0	0	0	0	0	0	0	0	0	0	0	0	0	0	0	0
*tetC*	0	0	0	0	0	0	0	0	0	0	0	0	0	0	0	0	0	0
*cat1*	0	0	3	3	2	4	2	1	0	1	16	0	0	0	0	0	0	0
*blaCTX*	0	2	3	0	1	1	1	0	0	1	9	0	3	4	1	0	0	8
*blaTEM*	1	2	2	3	1	1	1	1	1	0	13	0	3	2	0	0	0	5
*blaSHV*	1	0	5	3	0	2	2	1	0	0	14	0	2	2	1	1	0	6
*ermA*	0	0	0	0	0	0	0	0	0	0	0	0	0	0	0	0	0	0
*mcr1*	0	0	0	0	0	0	0	0	0	0	0	0	0	0	0	0	0	0
*int1*	1	4	9	3	4	6	2	1	1	1	32	0	2	1	0	0	0	3
*int2*	0	0	0	0	0	0	0	0	0	0	0	0	0	0	0	0	0	0
*int3*	0	0	0	0	0	0	0	0	0	0	0	0	0	0	0	0	0	0

*DEC* diarrhoeagenic *E. coli*, *EPEC* enteropathogenic *E. coli*, *ETEC* enterotoxigenic *E. coli*, *EAEC* enteroaggregative *E. coli*, *EHEC* enterohemorrhagic *E. coli*, *EIEC* enteroinvasive *E. coli*

**Table 3:** The association between DEC pathotypes and antimicrobial resistance gene; odds ratio (OR) (95% confidence interval)

AMR enes	EPEC		ETEC		EAEC		EIEC		EHEC	
	C	A	C	A	C	A	C	A	C	A
*qnrB*	6.9 (2.38–20.30)	0.0 (inf- inf)	6.8 (1.26–36.84)	inf. (inf- inf)	25.7 (4.66–142.14)	0.0 (inf- inf)	1.4 (0.16–14.10)	–	0.0 (inf- inf)	0.0 (inf- inf)
*qnrS*	8.3 (2.99–23.52)	8.7 (1.10–68.70)	5.5 (1.03–29.43)	31.3 (3.23–303.96)	5.7 (1.31–25.29)	0.0 (inf- inf)	3.4 (0.54–22.21)	–	0.9 (0.11–8.76)	0.0 (inf- inf)
*aac(6′)-Ib*	15.1 (5.24–43.89)	2.6 (0.25–27.29)	2.2 (0.39–13.31)	7.7 (0.65–92.05)	5.0 (1.16–21.99)	inf. (inf- inf)	7.3 (1.15–46.87)	–	2.2 (0.39–13.31)	0.0 (inf- inf)
*sulI*	36.0 ((9.71–133.99)	8.7 (1.10–68.70)	11.7 (1.32–103.96)	0.0 (inf- inf)	7.1 (1.37–37.20)	inf. (inf- inf)	3.2 (0.53–20.49)	–	4.5 (0.79–25.70)	0.0 (inf- inf)
*sulII*	10.3 (3.87–27.62)	10.6 (2.22–50.77)	3.5 (0.67–18.38)	3.1 (0.31–32.42)	6.3 (1.41–28.33)	inf. (inf- inf)	5.3 (0.84–33.47)	–	7.3 (1.27–42.52)	inf. (inf- inf)
*sulIII*	22.8 (2.54–205.95)	5.7 (0.85–38.54)	4.4 (0.44–45.88)	20.6 (2.46–173.35)	3.1 (0.32–30.69)	23.7 (1.25–452.35)	0.0 (inf- inf)	–	0.0 (inf- inf)	0.0 (inf- inf)
*dhfrI*	6.0 (2.28–16.17)	3.9 (0.33–47.27)	4.5 (0.86–24.25)	0.0 (inf- inf)	16.1 (3.02–86.53)	-inf. (inf- inf)	6.9 (1.09–44.08)	–	4.5 (0.86–24.25)	0.0 (inf- inf)
aac*(3)-IV*	4.2 (0.98–18.23)	–	9.2 (1.40–60.97)	–	2.2 (0.24–20.51)	–	3.9 (0.39–40.37)	–	0.0 (inf- inf)	–
*aadB*	1.2 (0.12–12.57)	0.0 (inf- inf)	7.6 (0.67–86.67)	inf. (inf- inf)	5.3 (0.49–58.12)	0.0 (inf- inf)	0.0 (inf- inf)	–	7.6 (0.67–86.67)	–
*tetA*	21.1 (4.14–107.68)	8.7 (1.10–68.70)	0.0 (inf- inf)	31.3 (3.23–303.96)	4.4 (0.77–25.76)	0.0 (inf- inf)	9.1 (1.33–63.02)	–	2.4 (0.25–22.82)	0.0 (inf- inf)
*tetD*	1.9 (0.33–11.21)	–	0.0 (inf- inf)	–	3.1 (0.32–30.69)	–	19.0 (2.45–147.34)	–	4.4 (0.44–45.88)	–
*tetB*	10.3 (2.81–37.99)	17.6 (1.46–211.86)	5.3 (0.87–32.92)	0.0 (inf- inf)	3.5 (0.62–19.68)	48.5 (2.17–1082.45)	0.0 (inf- inf)	–	1.9 (0.21–18.01)	0.0 (inf- inf)
*catI*	1.2 (0.38–4.38)	0.0 (inf- inf)	8.0 (1.46–43.84)	-inf. (inf- inf)	15.7 (3.31–75.02)	0.0 (inf- inf)	12.1 (1.85–79.38	–	8.0 (1.46–43.84)	0.0 (inf- inf)
*blaCTX*	9.4 (2.17–40.78)	4.6 (0.98–21.67)	0.0 (inf- inf)	72.8 (6.81–778.05)	5.1 (0.87–30.30)	-inf. (inf- inf)	3.4 (0.34–34.49)	–	2.7 (0.28–26.22)	0.0 (inf- inf)
*blaSHV*	3.3 (1.04–10.69)	3.3 (0.57–19.65)	5.3 (2.00–14.14)	12.1 (1.64–89.92)	2.8 (0.52–15.79)	-inf. (inf- inf)	2.0 (0.21–19.46)	–	9.6 (1.73–53.63)	-inf. (inf- inf)
*blaTEM*	3.8 (1.17–12.72)	14.5 (2.13–98.54)	10.7 (1.90–60.15)	20.6 (2.46–173.35)	11.7 (2.51–55.16)	0.0 (inf- inf)	2.2 (0.23–21.40)	–	1.7 (0.19–16.25)	0.0 (inf- inf)
*Int1*	8.1 (3.12–21.05)	5.7 (0.85–38.54)	3.0 (0.58–15.87)	20.6 (2.46–173.35)	inf. (inf- inf)	23.7 (1.25–452.35)	inf. (inf- inf)	–	3.0 (0.58–15.87)	0.0 (inf- inf)

*OR* odds ratio, *inf* infinity, *C* children, *A* adults

Unlike the strains isolated from children, only 73.68% (*n* = 14) of the DEC strains isolated from adults harbored one or more AMR genes. No AMR gene was detected in remaining 26.31% of strains. None of the isolates harbored *qnrA*, *qnrC*, *qepA*, *tetD*, *tetE*, *tetC*, *tetY*, *ermA*, *mcr1*, *int2*, and *int3* genes. Atypical EPEC strains harbored utmost classes of AMR genes than other DEC pathotypes (Tables 2 and 3).

### Antimicrobial susceptibility testing

Most of the DEC isolates phenotypically exhibited resistance for ceftriaxone [*n* = 33 (49.2%)] followed by co-trimoxazole [*n* = 29 (43.2%)], tetracycline [*n* = 24 (35.8%)], levofloxacin [*n* = 22 (32.8%)], ciprofloxacin [*n* = 20 (29.8%)], ceftazidime [*n* = 18 (26.2%)], gentamicin [*n* = 13 (19.4%)], chloramphenicol [*n* = 9 (13.4%)], amikacin [*n* = 5 (7.4%)], and cefoperazone-sulbactam [*n* = 5 (7.4%)] (Table 4). Four types of pattern were observed while correlating phenotypic and genotypic resistance pattern of the DEC isolates (Table 5).

**Table 4:** Antibiogram of DEC isolates (*n* = 67)

Antimicrobial class	Antimicrobials	Resistance	Intermediate	Sensitivity
β-lactam	Ceftriaxone	33 (49.2%)	1 (1.4%)	33 (49.2%)
	Ceftazidime	18 (26.8%)	10 (14.9%)	39 (58.2%)
	Cefoperazone-sulbactam	5 (7.4%)	0 (0.0%)	62 (92.5%)
Trimethoprim and sulfonamides	Co-trimoxazole	29 (43.2%)	1 (1.4%)	37 (55.2%)
Tetracycline	Tetracycline	24 (35.8%)	0 (0.0%)	43 (64.1%)
Quinolones	Levofloxacin	22 (32.8%)	3 (4.4%)	42 (62.6%)
	Ciprofloxacin	20 (29.8%)	11 (16.4%)	36 (53.7%)
Aminoglycosides	Gentamicin	13 (19.4%)	2 (2.9%)	52 (77.6%)
	Amikacin	5 (7.4%)	1 (1.4%)	61 (91%)
Phenicol	Chloramphenicol	9 (13.4%)	2 (2.9%)	56 (83.5%)

*DEC* diarrhoeagenic *E.coli*

**Table 5:** Correlation between phenotypic and genotypic resistance pattern of the DEC isolates (*n* = 67)

Antimicrobial class	Antimicrobials	AMR genes detected	Pattern 1	Pattern 2	Pattern 3	Pattern 4
Quinolones	Ciprofloxacin and levofloxacin	*qnrB*, *qnrS*, *aac(6′)-Ib*	13 (19.4%)	20 (29.8%)	9 (13.4%)	25 (37.3%)
Aminoglycosides	Amikacin and gentamicin	*aac3*, *aadB*	42 (62.6%)	9 (13.4%)	9 (13.4%)	7 (10.29%)
Tetracycline	Tetracycline	*tetA, tetB. tetD*	25 (37.3%)	13 (19.4%)	18 (23.8%)	11 (16.4%)
Phenicols	Chloramphenicol	*cat1*	44 (65.6%)	6 (8.9%)	12 (17.9)	5 (7.4%)
β-lactam	Ceftazidime, ceftriaxone, and cefoperazone-sulbactam	*blaCTX*, *blaTEM*, *blaSHV*	15 (22.5%)	16 (23.8%)	13 (19.4%)	23 (34.3%)
Trimethoprim and sulfonamide	Co-trimoxazole	*sul1*, *sulII*, *sulIII*, *dhfr1*	9 (13.4%)	1 (1.4%)	28 (41.7%)	29 (43.2%)

Pattern 1: No resistance gene(s) detected and phenotypically sensitive

Pattern 2: No resistance gene(s) detected but phenotypically resistant

Pattern 3: Resistance gene(s) detected but phenotypically sensitive

Pattern 4: Resistance gene(s) detected and phenotypically resistant

## Discussion

DEC infection was the most common among children as well as adults, and atypical EPEC infection was high in number followed by typical EPEC in children whereas in adults only atypical EPEC infection was detected (Table 1). Atypical EPEC is said to harbor only *eaeA* gene without EPEC adherence factor (EAF) plasmid (absence of *bfpA* gene), whereas in typical EPEC both the genes *eaeA* and *bfpA* are present. In our study interestingly, among the atypical EPEC detected, most were found to harbor *bfpA* gene alone and very few strains were positive for the *eaeA* gene. Variations in the distribution of atypical EPEC have been documented in earlier studies [19–24]. Atypical EPEC strain harboring *eaeA* gene without *bfpA* gene was detected in the studies conducted in Brazil, North-West Italy, and Melbourne [19–21]. A study from Iraq documented only atypical EPEC harboring *bfpA* without *eaeA* gene [22]. In a study conducted in Iran for both types of atypical EPEC, those harboring only *eaeA* and those harboring only *bfpA* were detected [23]. Another study from India documented the isolation of atypical EPEC (harboring only *eaeA* gene) from children with diarrhea and atypical EPEC (harboring only *bfpA* gene) from children without diarrhea in control group [24]. Hence, this indicates a mosaic distribution of the EPEC types with the atypical form emerging in many countries especially among the pediatric age group.

ETEC infection was common in children and adults next to EPEC infection. ETEC strains detected in children harbored only *lt* gene whereas in adults ETEC strains (*n* = 3) harbored *lt* and *st* and one strain of ETEC with only *st* gene was detected (Table 1). The reason for this strain variation among the adults and children is not so clear worldwide. Earlier study from India has documented no ETEC from children and adult [25]. ETEC is most commonly associated with traveler’s diarrhea [4].

In our study, EAEC was detected in children (11.1%) as well as in adults (2%) (Table 1). In recent years, EAEC has emerged as diarrhoeal agent causing acute and chronic diarrhea in all age groups though it was once known to infect primarily newborns and immunocompromised patients [4]. Studies conducted in Mexico, Bolivia, Nicaragua, and Myanmar found that EAEC was the most common among all the DEC pathotypes [14, 26–28]. On the contrary, in our study, EPEC was the most common, similar to the findings of the studies conducted in Israel, Norway [29, 30]. Studies from India indicate a lower prevalence rate of EAEC [24, 31].

STEC was detected in four children and one adult. Since 2006, there have been numerous outbreaks in the USA, Germany, and France due to STEC infection, most of these outbreaks were associated with packed or readymade food items, e.g., outbreak due to raw clover sprouts, and romaine lettuce [32]. STEC infection was less in both children and adults, and similar findings were documented in Kenya (0.2%), Nicaragua (2.1%), and Brazil (0.5%) [33–35].

Similar to STEC, EIEC detected were less in number but only from children and none from adults (Table 1). Studies conducted in Gabon and South Western Nigeria reported that no EIEC could be detected in children [36, 37]. While earlier studies documented that EIEC infection was most common in developing and underdeveloped countries [4]. The reduced detection rate of EIEC infection in children and adults may be reflective of a changing ecology of the EIEC pathotypes because of improvement in the personal hygiene, safe food practice, and proper decontamination of drinking water [38, 39].

Apart from infection with a single pathotype, various combinations of DEC co-infection were observed in both children (*n* = 3) and adults (*n* = 1) (Table 1). Combination of three DEC strains was detected in a co-infection in a study conducted in Nicaragua (ETEC, atypical EPEC, EAEC) [34]. While a study conducted in South Western Nigeria reported a combination of four DEC strains [EHEC + EPEC + ETEC (*LT*) + EAEC] [40]. Co-infection of atypical EPEC was observed maximum in number than other DEC pathotypes.

The number of hybrid DEC strains observed in children (*n* = 3) was relatively more compared to that of adults (*n* = 1) (Table 1). About 1% (DAEC-EAEC strain (*n* = 2), atypical EPEC-ETEC (*n* = 1)) of children were infected with hybrid DEC strains in Mexico [41]. In another study conducted by Dutta et al. in Kolkata, India, an EPEC-ETEC hybrid strain was isolated from a child with acute diarrhea [42]. Increasing trends in the hybrid phenomenon between EAEC and EHEC strains was documented elsewhere [43–45]. A hybrid strain of ETEC and EHEC also reported from Finland [46]. A study from Brazil reported nine hybrid strains (EAEC harboring UPEC virulence marker) from patients with bacteremia and urinary tract infection (UTI) [47]. Hence, this clearly implies that such hybrid strains have become a common phenomenon among the pathogenic *E. coli* and are not restricted only to the DEC due to HGT. They emerge as potential outbreak agents in recent years leading to the high cause of morbidity and mortality. In addition, the increased severity of infection was noticed among the people who are infected with hybrid DEC strains and in DEC co-infections [44].

In children, all the DEC strains isolated were resistant to at least one class of antibiotic and 14 out of 19 DEC strains from adults harbored resistance genes for one or more class of antibiotics (Table 2). The EPEC strains were found to harbor more number of resistance genes of various class of antimicrobial agents followed by EAEC and ETEC. Since 1960, trimethoprim and sulphonamides have been one of the WHO’s essential medicines, being easily affordable and very effective with a broad spectrum of activity against wide range of infection like diarrhea, cholera, UTI, and other extraintestinal infections. Recent studies have documented that trimethoprim and sulphonamide resistance genes are widespread being plasmid-borne, harbored by the bacteria inhabiting the aquatic bodies and in *E. coli* present in bio-fertilizers (cow dung) used in the agriculture as well as poultry farms [48]. These resistance genes are capable of circulating among the bacterial community through HGT. A transconjugation study conducted by Shuyu et al*.* showed that propagation of *sul* plasmid are majorly associated with incompatibility (IncF) replicons types (IncFI in *sulI* and IncFII in *sulII*) leading to resistance among the *E. coli* strains isolated from human stool specimen, and they also observed the co-transfer of other resistance genes [49]. Another transconjugation study conducted by Margarita Trobos et al. in *E. coli* strains found that *sul2* gene was transferred along with *blaTEM* in human [50]. Majority of the DEC strains in our study harbored the trimethoprim and sulphonamides resistance genes (Table 2).

A major resistance mechanism of quinolone resistance in the *Enterobacteriaceae* is plasmid-mediated (PMQR). The most common PMQR resistance genes were *qnrB*, *qnrS*, and *aac(6′)-Ib.* In children, all the pathotypes detected harbored either *qnrS* or *qnrB* genes or both, whereas, in adults, *qnrS* or *qnrB* was detected only in ETEC and atypical EPEC pathotypes (Table 1). A study from Spain reported *qnrA* and *qnrS* genes in their DEC strains [51]. A study from Ahmedabad, India, found that 64.7% of the ETEC strains only harbored *aac(6′)-Ib-cr* gene without *qnr* genes [52]. These studies suggest that dissemination of *qnr* resistance genes among these DEC is variable.

Following sulphonamides and quinolone, resistance to β-lactams (plasmid-borne genes) was detected to be high in our study. In a study conducted by Ghorbani-Dalini et al., 96.3% (*n* = 52) of DEC strains were found to harbor resistance genes to these antibiotics, *blaTEM* was positive in 83.33%, *blaSHV* in 31.48%, and *blaCTX-M* in 20.37% of adults [53]. In our study, majority of the DEC strains isolated from adults harbored *blaCTX-M* gene (57.1%) while in children, the *blaSHV* gene (29.1%) was more common (Table 2). In recent years, bla*CTX* has been the most predominant gene detected in *E. coli* in different parts of the world, particularly bla*CTX-*M-15 [54].

The frequency of aminoglycoside and chloramphenicol resistance varied among the DEC strains [55]. With reference to chloramphenicol resistance among *E. coli*, the major mechanism was inactivation of drugs by chloramphenicol acetyltransferase (CAT). *cat1* and *cat2* genes were most commonly distributed in *E. coli* strains worldwide since plasmid-borne, irrespective of the source of isolation [56, 57]. A study conducted by Yoo et al. found that low selective pressure was required for the transfer of chloramphenicol resistance genes among the bacteria isolated from aquatic regions [58]. This possibly indicates that such resistance genes and other AMR genes can be easily transferred by means of HGT as well leading to co-resistance.

Similar to aminoglycoside and chloramphenicol, tetracycline resistance genes are present in the plasmids harboring other drug resistance genes which can easily get transferred from one organism to the other by means of HGT, thus exhibiting the phenomenon of co-resistance to multiple antibiotics [59]. Due to broad-spectrum activity of tetracycline, it is widely used in veterinary practice to treat or prevent infections either through drinking water or feed [60]. A study from Ahmedabad, India, detected only *tetA*, *tetB*, and *tetE* genes without *tetC*, *tetD*, and *tetY* in the clinical isolates of ETEC in an outbreak [52]. We could not detect any *tetC*, *tetE*, or *tetY* genes in our strains (Table 2).

Many strains that harbored resistance genes were sensitive by phenotypic method which clearly implicates that these strains do not express the resistant genotype but act as a reservoir for these resistance genes. Meanwhile, resistance genes could not be detected in a few strains though they were phenotypically resistant, this could be due to the presence of other mechanisms of causing AMR such as efflux pumps.

Only class 1 integron (*int1* gene) was detected in DEC stains isolated from both children and adults in our study (Table 2). A similar study from Iran reported only *int1* and *int2* genes while none of the DEC strains harbored *int3* gene [61]. Studies from India also reported the presence of *int1* and *int2* genes in various DEC pathotypes [52, 62]. Low prevalence of class 2 integron (*int2* gene) and very rarely class 3 integron (*int 3* genes) have been reported so far among these strains. Class 1 integron was primarily linked with antibiotic resistance among these strains. Next to conjugative plasmid, the newly emerged *sulIII* gene is carried out in class 1 integron [51]. Role of class 1 integron is predominant in spreading the multiple drug resistant genes among these DEC strains despite the emergence of class 2 and 3 integrons.

## Conclusion

We observed that DEC is a potential diarrhoeal agent compared to other enteric bacterial pathogens in both children and adults in this study population. Atypical EPEC was most commonly encountered among DEC pathotypes. The emergence of atypical and DEC hybrid strains emphasize the importance of mobile genetic elements. Most of these DEC isolates were resistant to more than one antimicrobial agents and harbored integron genes, thus illustrating the importance of phenomenon of HGT arising due to the selective pressure of antibiotics. Thus, implementation of regulated use of antibiotics is the need of the hour in view of the availability of many antibiotics over the counter without the need of prescription, not completing the course of antibiotics, consumption of antibiotics when not indicated, and usage of antibiotics in animal feed. A surveillance network is thus needed and a mandatory reporting system to monitor these DEC strains and their AMR pattern for the effective control of diarrhoeal diseases.

## Additional files

Additional file 1: **Tables S1.** and **S2.** The details of targeted genes and primer sequences. (DOCX 25 kb)

Additional file 2: **Tables S3.** and **S4.** Correlation of DEC infection rate with age groups. (DOCX 16 kb)

## Abbreviations

AMR: Antimicrobial resistance
bp: Base pair
CAT: Chloramphenicol acetyltransferase
CDEC: Cell-detaching *E. coli*
*CFN*: Cytotoxic necrotizing factors
CIs: Confidence intervals
DEC: Diarrhoeagenic *Escherichia coli*
DNA: Deoxyribonucleic acid
EAEC: Enteroaggregative *E. coli*
EAF: EPEC adherence factor
EHEC: Enterohemorrhagic *E. coli*
EIEC: Enteroinvasive *E. coli*
EPEC: Enteropathogenic *E. coli*
ETEC: Enterotoxigenic *E. coli*
HGT: Horizontal gene transfer
HUS: Hemolytic-uremic syndrome
IQR: Interquartile range
LB: Luria-Bertani broth
NCBI: National Center for Biotechnology Information
NTEC: Necrotoxic *E. coli*
OR: Odds ratio
PMQR: Plasmid-mediated quinolone resistance
SD: Stranded deviation
STEC: Shiga toxin-producing *E. coli*
UTI: Urinary tract infection
