# Supplementary material for: A study of virulence and antimicrobial resistance pattern in diarrhoeagenic Escherichia coli isolated from diarrhoeal stool specimens from children and adults in a tertiary hospital, Puducherry, India
Source: J Health Popul Nutr. 2018 Jul 13;37:17. doi: 10.1186/s41043-018-0147-z (PMC6045864; doi:10.1186/s41043-018-0147-z)
Supplement: Supplementary file 1 — Tables S1. and S2. The details of targeted genes and primer sequences. (DOCX 25 kb) [file 41043_2018_147_MOESM1_ESM.docx]

**Table S1. PCR Primers used for the detection of virulence genes of DEC isolates in this study**

| **S.No** | **DEC Pathotypes** | **Genes** | **Primers**  **(5⇨3)** | **Product size (bp)** | **Reference** |
| --- | --- | --- | --- | --- | --- |
|  | EPEC | *eaeA*  *(attaching and effacing)* | F: GACCCGGCACAAGCATAAGC  R: CCACCTGCAGCAACAAGAGG | 384 | Pérez C, Gómez-Duarte O, Arias M. Diarrheagenic Escherichia coli in Children from Costa Rica. Am J Trop Med Hyg. 2010;83(2):292-297. |
|  |  | *bfpA*  *(bundle-forming pilus)* | F: AATGGTGCTTGCGCTTGCTGC  R:GCCGCTTTATCCAACCTGGTA | 324 |  |
|  | ETEC | *lt*  *(heat-labile toxin)* | F:GGCGACAGATTATACCGTGC  R:CGGTCTCTATATTCCCTGTT | 450 |  |
|  |  | *st*  *(heat-stable toxin)* | F:ATTTTTCTTTCTGTATTGTCTT  R:CACCCGGTACAAGCAGGATT | 190 |  |
|  | EHEC | *stx1*  *(Shiga toxins 1)* | F:CTGGATTTAATGTCGCATAGTG  R:AGAACGCCCACTGAGATCATC | 150 |  |
|  |  | *stx2*  *(Shiga toxins 2)* | F:GGCACTGTCTGAAACTGCTCC  R:TCGCCAGTTATCTGACATTCTG | 255 |  |
|  | EIEC | *ial*  *(invasive-associated locus)* | F:GGTATGATGATGATGAGTCCA  R:GGAGGCCAACAATTATTTCC | 650 |  |
|  | EAEC | *aggR*  *(transcriptional regulator)* | F: GTATACACAAAAGAAGGAAGC  R: ACAGAATCGTCAGCATCAGC | 254 |  |
|  | CDEC/  NTEC | *CFN1*  *(Cytotoxic Necrotizing Factors-1)* | F: GAACTTATTAAGGATAGT  R: CATTATTTATAACGCTG | 543 | Orden JA, Ruiz-Santa-Quiteria JA, Cid D, García S, de la Fuente R. Prevalence and characteristics of necrotoxigenic Escherichia coli (NTEC) strains isolated from diarrhoeic dairy calves. Vet Microbiol. 1999;66(4):265-273. |
|  |  | *CFN2*  *(Cytotoxic Necrotizing Factors-2)* | F: AATCTAATTAAAGAGAAC  R: CATGCTTTGTATATCTA | 543 |  |

**DEC**- Diarrheagenic *E. coli*; **EPEC**- Enteropathogenic *E. coli*; **ETEC**- Enterotoxigenic *E. coli*; **EAEC**- Enteroaggregative *E. coli*; **EHEC**- Enterohaemorrhagic *E. coli*; **EIEC**- Enteroinvasive *E. coli*; **NTEC**- Necrotoxigenic *E. coli* **; CDEC**- Cell Detaching *E. coli*. bp- base pairs.

**Table S2. PCR primers used for the detection of antimicrobial resistance genes**

| **S.No** | **Anti-**  **microbial**  **class** | **Genes** | **Primers**  **(5⇨3)** | **Product**  **size**  **(bp)** | **Reference** |
| --- | --- | --- | --- | --- | --- |
|  | Quinolone | *qnrA* | F: ATTTCTCACGCCAGGATTTG  R: GATCGGCAAAGGTTAGGTCA | 516 | Kim HB, Park CH, Kim CJ, Kim EC, Jacoby GA, Hooper DC. Prevalence of Plasmid-Mediated Quinolone Resistance Determinants over a 9-Year Period. Antimicrob Agents Chemother. 2009:53(2): 639-645 |
|  |  | *qnrB* | F: GATCGTGAAAGCCAGAAAGG  R: ATGAGCAACGATGCCTGGTA | 476 |  |
|  |  | *qnrS* | F: GCAAGTTCATTGAACAGGGT  R: TCTAAACCGTCGAGTTCGGCG | 428 |  |
|  |  | *qnrC* | F: GGGTTGTACATTTATTGAATCG  R: CACCTACCCATTTATTTTCA | 307 |  |
|  |  | *qepA* | F: AACTGCTTGAGCCCGTAGAT  R: GTCTACGCCATGGACCTCAC | 482 |  |
|  |  | *aac(6′)-Ib* | F: TTGCGATGCTCTATGAGTGGCTA  R: CTCGAATGCCTGGCGTGTTT | 596 |  |
|  | Sulphonamides | *sulI* | F: TTCGGCATTCTGAATCTCAC  R: ATGATCTAACCCTCGGTCTC | 822 | Maynard C, Fairbrother JM, Bekal S, Sanschagrin F, Levesque RC, Brousseau R et al. Antimicrobial Resistance Genes in Enterotoxigenic Escherichia coli O149:K91 Isolates Obtained over a 23-Year Period from Pigs. Antimicrob Agents Chemother. 2003:47(10):3214-3221.  and  Boerlin P, Travis R, Gyles CL, Smith RR, Janecko N, Lim H. Antimicrobial Resistance and Virulence Genes of Escherichia coli Isolates from Swine in Ontario. Appl. Environ. Microbiol. 2005:71(11): 6753-6761 |
|  |  | *sulII* | F: CGGCATCGTCAACATAACC  R: GTGTGCGGATGAAGTCAG | 722 |  |
|  |  | *sulIII* | F: GAGCAAGATTTTTGGAATCG  R: CATCTGCAGCTAACCTAGGGCTTTGGA | 880 |  |
|  | Trimethoprim | *dhfrI* | F: AAGAATGGAGTTATCGGGAATG  R: GGGTAAAAACTGGCCTAAAATTG | 391 |  |
|  | Aminoglycoside | aac*(3)-IV* | F: GTGTGCTGCTGGTCCACAGC  R: AGTTGACCCAGGGCTGTCG | 627 |  |
|  |  | *aadB* | F: TCCAGAACCTTGACCGAAC  R: GCAAGACCTCAACCTTTTCC | 700 |  |
|  | Tetracycline | *tetA* | F: GTGAAACCCAACATACCCC  R: GAAGGCAAGCAGGATGTAG | 888 |  |
|  |  | *tetY* | F: ACCGCACTCATTGTTGTC  R: TTCCAAGCAGCAACACAC | 823 |  |
|  |  | *tetD* | F: TGGGCAGATGGTCAGATAAG  R: CAGCACACCCTGTAGTTTTC | 827 |  |
|  |  | *tetE* | F: TTAATGGCAACAGCCAGC  R: TCCATACCCATCCATTCCAC | 853 |  |
|  |  | *tetC* | F: ACTTGGAGCCACTATCGAC  R: CTACAATCCATGCCAACCC | 881 |  |
|  |  | *tetB* | F: CCTTATCATGCCAGTCTTGC  R: ACTGCCGTTTTTTCGCC | 774 |  |
|  | Chloramphenicol | *catI* | F: AGTTGCTCAATGTACCTATAACC  F: ACACTTTGCCCTTTATCGTC | 547 |  |
|  | β lactams | *blaCTX* | F: ATGTGCAGYACCAGTAARGT  R: TGGGTRAARTARGTSACCAGA | 1018 | Mandal J, Sangeetha V, Nivedithadivya D, Das A, Parija S. Characterization of Extended-spectrum β-lactamase producing Clinical Isolates of Shigellaflexneri. J Health PopulNutr. 2013;31(3):405-408. |
|  |  | *blaSHV* | F: ATTTGTCGCTTCTTTACTCGC  R: TTTATGGCGTTACCTTTGACC | 1076 |  |
|  |  | *blaTEM* | F: ATAAAATTCTTGAAGACGAAA  R: GACAGTTACCAATGCTTAATC | 544 |  |
|  | Macrolide | *ermA* | F: TCTAAAAAGCATGTAAAAGAAA  R: CGATACTTTTTGTAGTCCTTC | 533 | Nguyen MCP, Woerther PL, Bouvet M, Andremont A, Leclercq R, Canu A. Escherichia coli as Reservoir for Macrolide Resistance Genes. Emerg Infect Dis. 2009:15(10): 1648-1650. |
|  | Colistin | *mcr1* | F:CGGTCAGTCCGTTTGTTC  R: CTTGGTCGGTCTGTAGGG | 309 | Elnahriry SS, Khalifaa HO, Solimana AM, Ahmeda AM, Husseinb AM, Shimamoto T. Emergence of Plasmid-Mediated Colistin Resistance Gene mcr-1 in a Clinical Escherichia coli Isolate from Egypt. Antimicrob. Agents Chemother. 2016: 60 (5):3249-3250 |
| 11. | **class 1 integrons** | *int1* | F: GGTCAAGGATCTGGATTTCG  R: ACATGCGTGTAAATCATCGTC | 436 | Kargar M, Mohammadalipour Z, Doosti A, Lorzadeh S, Nejad A J. High Prevalence of Class 1 to 3 Integrons Among Multidrug-Resistant Diarrheagenic Escherichia coli in Southwest of Iran. Osong Public Health Res Perspect 2014 5(4): 193-198. |
| 12. | **class 2integrons** | *int2* | F: CACGGATATGCGACAAAAAGG  R: TGTAGCAAACGAGTGACGAAATG | 788 |  |
| 13. | **class 3 integrons** | *int3* | F: AGTGGGTGGCGAATGAGTG  R: TGTTCTTGTATCGGCAGGTG | 600 |  |

bp – base pairs
