# Supplementary material for: A study of virulence and antimicrobial resistance pattern in diarrhoeagenic Escherichia coli isolated from diarrhoeal stool specimens from children and adults in a tertiary hospital, Puducherry, India
Source: J Health Popul Nutr. 2018 Jul 13;37:17. doi: 10.1186/s41043-018-0147-z (PMC6045864; doi:10.1186/s41043-018-0147-z)
Supplement: Supplementary file 2 — Tables S3. and S4. Correlation of DEC infection rate with age groups. (DOCX 16 kb) [file 41043_2018_147_MOESM2_ESM.docx]

**Table S3. Correlation of DEC infection rate with age groups (Children)**

| Correlation of DEC infection rate with age groups (Children) | | | |
| --- | --- | --- | --- |
| Age (months) expressed in median^*^ (IQR)^**^ – 12 (5.25-24) | | | |
| Age in Month | DEC Infection Positive  % | DEC Infection Negative  % | Total |
| 0to12 | 21 (17.5) | 41 (34.1) | 62 (51.6) |
| 13to24 | 20 (16.66) | 24 (20) | 44 (36.6) |
| 25to36 | 1 (0.83) | 0 (0.83) | 1 (0.833) |
| 37to48 | 1 (0.83) | 8 (6.6) | 9 (7.5) |
| 49to60 | 2 (1.66) | 2 (1.6) | 4 (3.3) |
| Total | 45 (37.5) | 75 (62.5) | 120 (100) |

*Age expressed in median(Non- Normally Distribution)

** IQR - interquartile range

**Table S4: Correlation of DEC infection rate with age groups (Adults)**

| Correlation of DEC infection rate with age groups (Adults) | | | |
| --- | --- | --- | --- |
| Mean [±SD^*^] Age - 44.74 [±15.24] | | | |
| Age in Year | DEC Infection Positive  % | DEC Infection Negative  % | Total  % |
| 18to24 | 1 (1) | 8 (8) | 9 (9) |
| 25to36 | 5 (5) | 20 (20) | 25 (25) |
| 37to48 | 5 (5) | 16 (16) | 21 (21) |
| 49to85 | 7 (7) | 38 (38) | 45 (45) |
| Total | 18 (18) | 82 (82) | 100 (100) |

* SD: Standard Deviation
